# Laparoscopic Removal of Gossypiboma

**DOI:** 10.1155/2015/317240

**Published:** 2015-09-16

**Authors:** Zeki Özsoy, Ismail Okan, Emin Daldal, Mehmet Fatih Dasıran, Yavuz Selim Angın, Mustafa Şahin

**Affiliations:** Department of General Surgery, Gaziosmanpasa University Faculty of Medicine, 60100 Tokat, Turkey

## Abstract

Gossypiboma is defined as a mass caused by foreign body reaction developed around the retained surgical item in the operative area. When diagnosed, it should be removed in symptomatic patients. Minimal invasive surgery should be planned for the removal of the retained item. The number of cases treated by laparoscopic approach is rare in the literature. We present a case of forty-year-old woman referred to emergency room with acute abdomen diagnosed as gossypiboma and treated successfully with laparoscopic surgery.

## 1. Introduction

Gossypiboma defines a mass lesion shaped by foreign body reaction developed around sponge and such substances retained in the operative area. The abdominal cavity is the largest inside space of the human body. The most frequently retained objects are the cotton sponges which are often retained in the abdomen during general surgery operations. Surgical items including needles, clamps, retractors, and drains are reported to be retained in the abdominal cavity. They can either stay asymptomatic for years or present with abscess/peritonitis symptoms. The presence of foreign body is suspected by radiological imaging. The foreign body should be removed when diagnosed in symptomatic patients [1, 2]. The process of removing can be managed with laparoscopy and endoscopy with the aid of ultrasonography as well as open surgery depending on the available resources and the experience of the caring team.

## 2. Case Report

**Figure 1:**
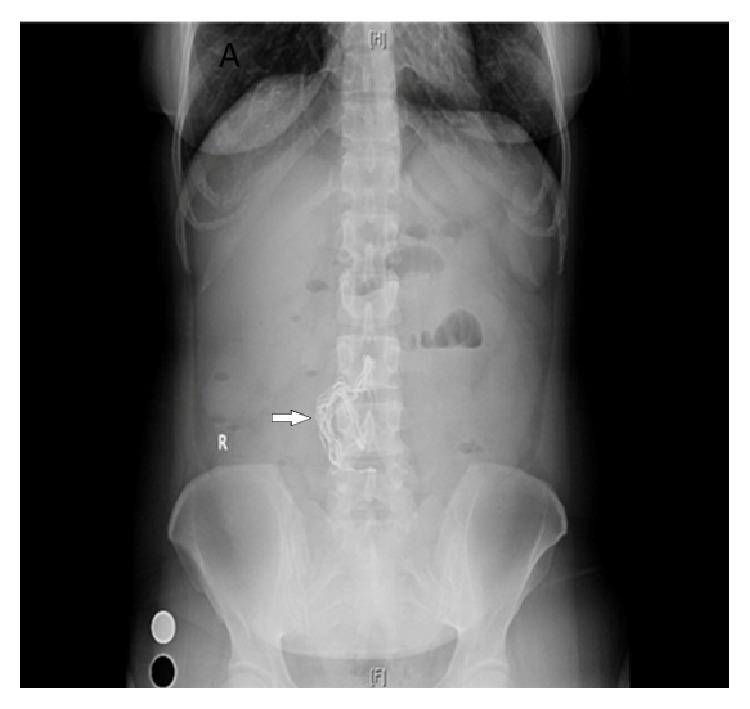
A direct abdominal radiography showed multiple opaque substances accumulated as strips in the mid-line.

A forty-year-old female patient was admitted to the emergency room due to abdominal pain and vomiting. She had no passage of gas and stool for two days. She had no fever and her vital findings were stable. Physical examination revealed a mass of eight centimeters in diameter palpated on the epigastric region. She had leucocytosis; the other blood analyses were normal. She had hysterectomy operation with Pfannenstiel incision four years ago and has been occasionally having abdominal pain after the operation. Air-fluid levels in a few intestinal loops and multiple opaque substances accumulated as a strip in the mid-line were noticed in the direct abdominal radiography (Figure 1).

**Figure 2:**
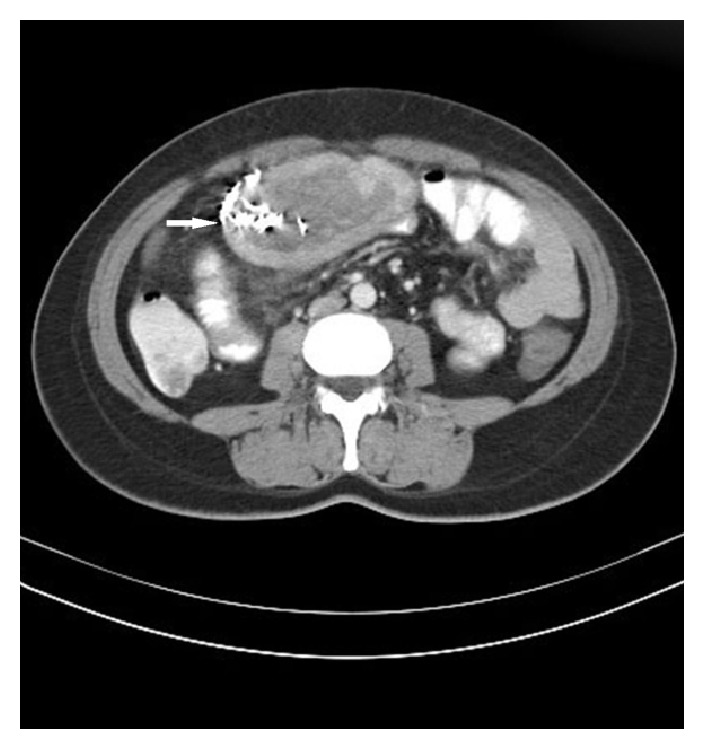
Gossypiboma in abdominal computed tomography.

The patient underwent contrast enhanced abdominal computed tomography (CT). A mass lesion suggesting gossypiboma among small bowel loops with dimensions of 107 × 60 mm in the level of L2–4 vertebrae, closely related with anterior abdominal wall, was detected. Inside the right half of the mass linear metallic hypodense artefacts and peripheral contrast enhancement were noticed (Figure 2).

**Figure 3:**
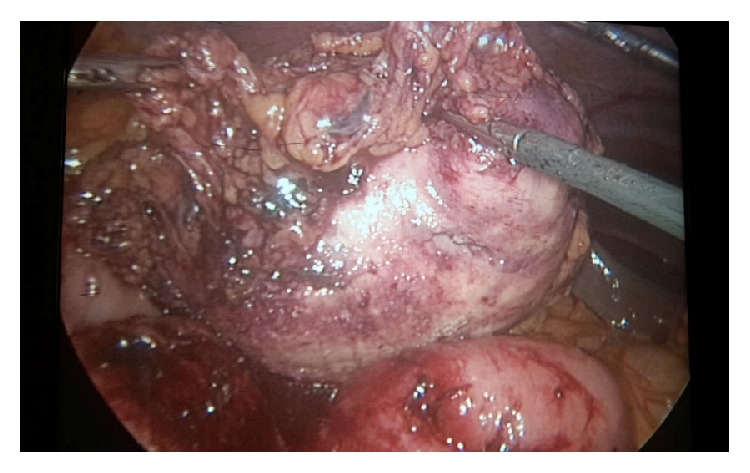
Laparoscopic approach.

The patient was hospitalized and submitted to the operation on the same day. Laparoscopic approach was preferred in the operation. 10 mm camera port was inserted under direct vision below the umbilicus. Three 5 mm working trocars were inserted from left inguinal region, left subcostal region, and epigastric region. The jejunal and ileal loops adherent to the mass were separated with sharp and blunt dissections (Figure 3).

**Figure 4:**
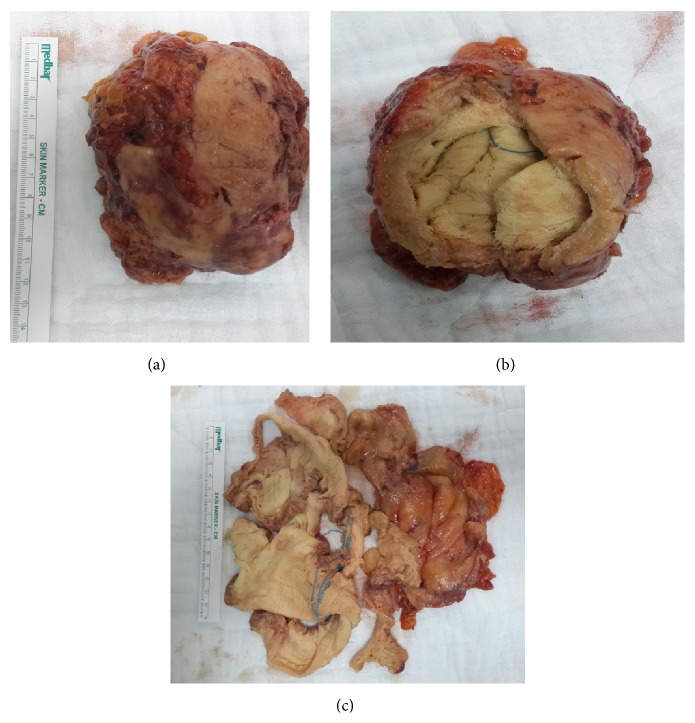
(a) Removed gossypiboma. (b) Approximately 1 cm thick capsule developed with the granulation tissue around the laparotomy pad. (c) Laparotomy pad with radiopaque markers.

The mass was removed with laparoscopic (tissue) extraction bag after dissection of the adhesions completely. The umbilical trocar incision was extended to 6 cm length for the removal of the mass (Figure 4(a)). When the capsule of the removed specimen was opened, the laparotomy pad was discovered with radiopaque markers (Figures 4(b) and 4(c)). The diagnosis was histopathologically confirmed. The operation lasted for three hours with minimal blood loss and minimal small intestine serosal lacerations. The postoperative period was uneventful and the patient was discharged from hospital on the 4th postoperative day. She has been followed up for eight months without any complication.

## 3. Discussion

Surgical items left in the patients' bodies after completion of the operation can lead to serious medical, legal, and social problems both for patient and surgeon [3]. It is estimated that retained surgical item is seen in laparotomies with a varying rate of 1/1000–1500, but legal concern and asymptomatic patients obscure the real incidence [4].

Factors associated with retained surgical items include emergency surgical procedures, unexpected changes in the course of operation, duration of the operation, excessive loss of blood, alteration of surgical team or nurses during the operation, and inexperienced and insufficient number of personnel. Moreover, there are some other factors which are related to the patient's condition such as obesity and female gender [5]. The majority of the reported cases are retained items in gynaecologic operations which is the reason why it is more commonly seen in female sex since the width of the surgical site and difficulty of exploration increase the risk of oversight of the sponges for obese patients.

The common items which are left during the operations are cotton sponges and laparotomy pads whose colours and patterns may change after contact with blood and other body fluids [1]. In addition to aforementioned items, surgical instruments may also be left in the abdomen due to the lack of the team concentration who undertake operations. Although the most common site for the retained objects is abdominal cavity, other sites of the body like thorax, breast, para neural site, cranium, spinal cord, vagina, neck, nose, tracheobronchial tree, and extremities may be involved.

The morbidity and mortality are low when noticed and removed in the early postoperative period. However, the longer it stays in the body, the more the complications rise. In the early postoperative period, the patient usually present septic findings, whereas it might be asymptomatic or present with vague symptoms. The retained foreign body causes histopathologically two types of foreign body reactions. In the first one, adhesion, encapsulation, and granulation take place with an aseptic fibrotic reaction around the foreign body [4]. In such cases the patient may not have apparent complaints and findings and it can be detected incidentally. The latter one is the exudative reaction which causes cyst and abscess formations [6]. In our case, approximately 1 cm thick capsule developed with the granulation tissue around the retained laparotomy pad during 4 years and it stayed asymptomatic. It is assumed that the laparotomy pad might have been used for removing the small bowels away from the surgical site. But our patient has reported that she had been having abdominal pain occasionally. In her last admission, severe abdominal pain and constipation complaints resolved with hydration and laxative treatment. Attached bowel loops to the mass found in exploration explained the intermittent nature of the complaints in our case. The retained foreign body may transmurally immigrate to stomach, duodenum, small intestine, large intestine, diaphragm, bladder, and vagina. After migration the patients either may be asymptomatic or can present with perforation, obstruction, bleeding, or fistula findings [1, 7–9].

It is difficult to diagnose retained items in asymptomatic patients. These items can sometimes be noticed incidentally in radiological imaging like X-ray, magnetic resonance images (MRI), CT, and ultrasonography (USG) [10]. Radiopaque markers found in foreign bodies help us to identify the mass. In USG, it is seen as a uniformly bounded mass, hyperechoic in the middle and hypoechoic in the sides, with intense acoustic shadow in the background [11]. In CT, a spongiform mass composed of air-filled holes among fibres of gauze and the contrast-holding fibrotic capsular appearance is typical [12]. Final diagnosis is reached in surgical exploration and after the pathological examination. In our case, the diagnosis has been made before surgery with the help of typical appearance and presence of radiopaque markers in both X-ray and CT. The presenting symptoms and the previous gynaecological operation raised the suspicion for gossypiboma. This mass can mimic the appearance of a tumour radiologically in some cases and it can also be misinterpreted as intra-abdominal hydatid cyst, soft tissue neoplasms, or abscess [4, 13, 14].

Once gossypiboma is diagnosed, the treatment is to remove the retained material. It can be done both with open or laparoscopic surgery, as well as endoscopy. A foreign body transmurally migrated to stomach or colon can be removed by endoscopy [15]. Laparoscopy has the advantages of shorter hospitalisation, less postoperative pain, and better cosmetic appearance. There are limited number of studies with laparoscopic approach in the gossypiboma treatment. Târcoveanu et al. used laparoscopic surgery to four (4) patients out of forty-eight (48) on whom they operated with intra-abdominal foreign body diagnosis in twenty (20) years. Only in one patient they converted to open surgery due to intense adhesion of foreign body to the stomach wall. At the end of the study they concluded that laparoscopy can be safely implemented in small, encapsulated sponge without complication [16]. Justo et al. detected the retained item as a gastrointestinal stromal tumour, after 34 years from their first operation due to peptic ulcer surgery. During laparoscopy, they noticed gossypiboma and removed the mass laparoscopically [17]. Sista et al. noticed gossypiboma in their 40-year-old case after 9 years from her elective caesarean section and removed the mass which was approximately 20 cm in diameter by minilaparotomy through Pfannenstiel incision. They concluded that less complications, shorter hospitalisation duration, and better cosmetic results could be reached with laparoscopic approach [18].

The main reason we preferred laparoscopic approach in this case is that diagnosis was clear and clinically and hemodynamically stable and the mass was uniformly bounded in the CT images. The mass has been put into the laparoscopic extraction bag and removed by minilaparotomy as the dimension of the mass was too large. We therefore believe that laparoscopy can contribute to patient satisfaction by providing less hospitalisation and better cosmetic appearance.

The important point to prevent the occurrence of gossypiboma is to take necessary actions, follow the operation procedure properly, and take necessary precautions into account throughout the operations. Some of the necessary precautions are to count all the equipment both at the beginning and at the end of the operation, to keep good record, to have radiological markers on sponges and pads, and to use scope if any doubt is present [15]. Nursing team should be warned about the counts during change of the shift. Surgeon should be informed about counting before the operation is finished and be sure that the used materials are complete.

## 4. Conclusion

Gossypiboma may be a life-threatening problem causing serious results. Differential diagnosis should be kept in mind in patients who had an operation background and in whom intra-abdominal mass was detected. Surgery should be planned in symptomatic patients since legal and medical problems may occur. Laparoscopic surgery provides the advantages of smaller incision, less pain, shorter hospitalisation, and reduced haemorrhage and infection ratios compared to open surgery. Laparoscopy can be safely implemented in appropriate cases by experienced surgeons. But it should not be forgotten that prevention is easier than treatment.

## Abbreviations

CT: Computed tomography
MRI: Magnetic resonance image
USG: Ultrasonography.
